# The Significance of Circulating Cell-Free DNA Markers in Chronic Hepatitis B Patients with Hepatocellular Carcinoma

**DOI:** 10.3390/pathogens10111524

**Published:** 2021-11-22

**Authors:** Alkistis Papatheodoridi, Nikolaos Karakousis, Panagiotis Lembessis, Antonios Chatzigeorgiou, George V. Papatheodoridis

**Affiliations:** 1 Department of Clinical Therapeutics, “Alexandra” General Hospital of Athens, Medical School of National, Kapodistrian University of Athens, 11528 Athens, Greece; alkistispapath@gmail.com; 2 Department of Physiology, Medical School of National, Kapodistrian University of Athens, 11527 Athens, Greece; karak2727@gmail.com (N.K.); lembessis@hotmail.com (P.L.); 3 Department of Gastroenterology, General Hospital of Athens “Laiko”, Medical School of National, Kapodistrian University of Athens, 11527 Athens, Greece; 4 Institute for Clinical Chemistry and Laboratory Medicine, University Clinic Carl Gustav Carus, Technische Universität Dresden, 01307 Dresden, Germany

**Keywords:** hepatitis B, hepatocellular carcinoma, cell-free DNA, nucleosomes, DNA integrity

## Abstract

(1) Background: Hepatocellular carcinoma (HCC) is the most serious complication of chronic hepatitis B (CHB). Recently, the detection of circulating cell-free (cf) DNA and nucleosomes has found numerous applications in oncology. This study aimed to examine the levels of serum cfDNA markers and nucleosomes in CHB patients with and without HCC and assess their potential association with HCC patients’ survival. (2) Methods: Nineteen patients with CHB and HCC and 38 matched patients with CHB without cancer development during 5 years of antiviral therapy were included. Stored serum samples were analyzed for cfDNA species, including the cfDNA concentration and levels of Alu115, Alu247, and nucleosomes. DNA integrity was expressed as the Alu247/Alu115 ratio. (3) Results: Compared to controls, HCC patients had higher median Alu247 levels (64.2 vs. 23.2 genomic equivalent, *p* = 0.004) and DNA integrity (1.0 vs. 0.7, *p* < 0.001) and a trend for a higher median cfDNA concentration (36.0 vs. 19.5 ng/mL, *p* = 0.064). Increased DNA integrity (Alu247/Alu115 > 1) was associated with an increased risk of death during the first year after HCC diagnosis (*p* = 0.016). (4) Conclusions: Levels of Alu247 and DNA integrity in serum cfDNA are elevated in CHB patients with HCC, whereas increased DNA integrity seems to be associated with a worse short-term prognosis in this setting.

## 1. Introduction

Hepatocellular carcinoma (HCC) is currently classified as the most common type of liver cancer [1]. Worldwide, most HCC cases are associated with chronic hepatitis B (CHB) and/or cirrhosis of any etiology [1]. Patients with CHB usually receive long-term oral antiviral therapy, achieving virological remission in almost all compliant cases and improving liver histological lesions and their overall outcome [2,3,4]. However, HCC may develop even in effectively treated patients and remains the only factor that affects liver-related mortality [5,6]. Therefore, the identification of HCC risk factors is of great importance in this setting.

Cell-free DNA (cfDNA) is free-floating genetic material found in the plasma, serum, and other body fluids [7]. Recently, cfDNA detection has found numerous applications in oncology, including early diagnosis, detection of minimal residual disease, evaluation of treatment response, and resistance [7,8,9,10]. Non-long terminal repeats, such as Alu elements, are overrepresented in cfDNA [11] and are often used for distinguishing necrotic cell death induced by tumorigenesis and apoptosis [12]. During apoptosis, shorter fragments of DNA are released in the circulation, allowing us to detect short Alu elements of 115 pair bases (bp) [13]. However, in cancer, the rate of necrotic death increases, resulting in increased concentration of longer fragments, such as Alu elements of 245 bp, in cfDNA [12,13,14,15]. Thus, the parameter of DNA integrity, defined as the ratio of longer fragments to shorter fragments, is usually elevated in cancer patients [14,15]. Elevated levels of total circulating nucleosomes have also been recorded in cancer patients because of increased cell turnover [16,17]. Nucleosomes consist of small DNA chains (147 bp) wrapped around a histone octamer and are suspected to carry information for the generation of new metastases in cancer; therefore, their concentration is frequently elevated in the plasma and serum of cancer patients [16,17]. 

The aim of this study was to compare the serum levels of these biomarkers in CHB patients with and without HCC and to assess their potential associations with the survival of patients with HCC.

## 2. Results

### 2.1. Patient Characteristics 

The main baseline characteristics of the 19 HCC cases and 38 CHB controls are presented in Table 1. The two groups did not differ significantly in most baseline characteristics. Compared to CHB controls, HCC cases had normal alanine aminotransferase (ALT) less frequently (2/19 or 13.3% vs. 15/38 or 44.1%, *p* = 0.037). The HCC risk according to the PAGE-B score was high or moderate in all HCC cases and CHB controls. The Barcelona Clinic Liver Cancer (BCLC) stage was A in 2 (10.5%), B in 15 (78.9%), and C in 2 (10.5%) patients with HCC. All patients with BCLC stage A underwent surgical resection, all patients with BCLC stage B were treated with transarterial chemoembolization, and all patients with BCLC stage C were treated with sorafenib.

### 2.2. Circulating cfDNA Species and cfDNA Integrity

**Figure 1:**
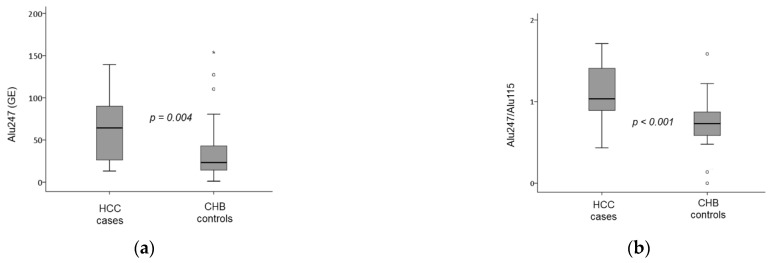
Levels of Alu247 (**a**) and the Alu 247/115 ratio (**b**) in HBeAg-negative chronic hepatitis B patients (CHB) with (HCC cases, n = 19) and without (Controls, n = 18) development of hepatocellular carcinoma (HCC). Box and whisker plots show median, 25th–75th percentile, and minimum–maximum values. Outliers are shown by “^o^” or “*”.

Circulating cfDNA species in HCC cases and CHB controls are presented in Table 2. The median cfDNA concentration tended to be higher in HCC cases than CHB controls, but the difference did not reach statistical significance (36.0 (94.0) vs. 19.5 (27.3) ng/mL, *p* = 0.064). The median Alu115 serum levels did not differ significantly between HCC cases and CHB controls (50.1 (68.2) vs. 33.9 (33), *p* = 0.139). However, the median levels of Alu247 were significantly higher in the HCC cases compared to CHB controls (64.2 (67.9) vs. 23.2 (29.7), *p* = 0.004) (Figure 1a). In addition, DNA integrity (Alu247/Alu115 ratio) was significantly higher in the group of HCC cases compared to CHB controls (1.0 (0.7) vs. 0.7 (0.3), *p* < 0.001) (Figure 1b). Similarly, increased DNA integrity (Alu247/Alu115 > 1) was observed significantly more frequently in HCC cases than CHB controls (57.9% vs. 15.8%, *p* = 0.001). The levels of nucleosomes were similar between the two groups. 

Since the proportion of increased DNA integrity differed significantly between HCC cases and CHB controls and was also associated with one-year mortality of HCC cases, as shown below, the associations between increased DNA integrity and other patient’ characteristics were determined (Table 3). There was no association between increased DNA integrity with any patient characteristic, except for a trend for probably a random association with platelet groups, as increased DNA integrity was observed relatively more frequently in patients with moderate platelet counts (100,000–199,999/mm^3^: 14/39 or 36%) than in patients with high (≥200,00/mm^3^: 1/6 or 17%) or low (<100,00/mm^3^: 0/10) platelet counts (*p* = 0.06). The proportion of increased DNA integrity was higher in patients with HCC (11/19 or 58%) vs. non-HCC patients with cirrhosis (1/14 or 7%, *p* = 0.004) or without cirrhosis (5/24 or 21%, *p* = 0.025) and similar between cirrhotic and non-cirrhotic patients with HCC (7/10 or 70% vs. 4/9 or 44%, *p* = 0.370) or without HCC (1/14 or 7% vs. 5/24 or 21%, *p* = 0.383). In patients with HCC, the median alfa-fetoprotein levels did not differ between cases with and without increased DNA integrity (340 (751) vs. 286 (2061) ng/mL, *p* = 0.717). 

### 2.3. Survival

**Figure 2:**
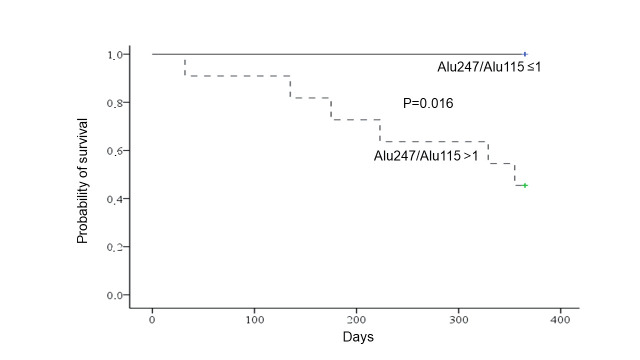
Probability of survival within the first year after diagnosis of hepatocellular carcinoma in 19 patients with chronic hepatitis B in relation to the presence of increased DNA integrity (Alu247/Alu115 ratio > 1). Log-rank test was used to assess statistical significance.

The median overall survival for HCC cases was 523 (821) days. Univariate Cox regression analysis for circulating cfDNA species and patients’ baseline characteristics including HCC BCLC stage did not reveal any factor to be significantly associated with HCC cases’ overall survival (Table 4). However, subgroup analyses for patients’ one-year mortality revealed that only DNA integrity was associated with an increased risk of death during the first year after HCC diagnosis (hazard ratio (HR: 2.46, 95% confidence interval (CI): 1.15–5.28; *p* = 0.020) (Table 4). Particularly, compared to patients without an increased Alu247/Alu115 ratio (>1) at baseline, those with an increased ratio were at increased risk of dying during the first year after HCC diagnosis (log-rank, *p* = 0.016) (Figure 2). DNA integrity remained an independent risk factor for patients’ one-year mortality, even after adjustment for HCC BCLC stage (HR: 2.19, 95% CI: 1.02–4.71; *p* = 0.043). 

## 3. Discussion

Liquid biopsy biomarkers, including circulating cfDNA and nucleosomes, have been recently associated with inflammation and malignancies and seem to represent promising markers for both early detection and monitoring the response to treatment of several cancers [8,17]. This is the first study attempting to evaluate the significance of circulating biomarkers, such as cfDNA, its components, and nucleosomes in CHB patients with HCC. In particular, we hypothesized that quantitative and qualitative alterations in serum cfDNA or nucleosomes may be implicated in hepatocarcinogenesis in CHB patients and potentially affect the prognosis after HCC development.

Our major finding was that DNA integrity, expressed as the ratio of longer DNA fragments to shorter DNA fragments (Alu247/Alu115), was higher, and increased DNA integrity (Alu247/Alu115 > 1) was more frequently observed in CHB patients with HCC compared to CHB patients without HCC during ≥5 years of follow-up in patients receiving therapy who were carefully matched for known HCC risk factors. Higher DNA integrity has been previously associated with several malignancies [14,15]. Interestingly, increased DNA integrity of cfDNA was associated with a higher risk of death during the first year after HCC diagnosis, although it was not associated with overall survival. DNA integrity has previously been associated with advanced tumor stages and poorer prognosis [18,19]. The association of increased DNA integrity with worse one-year overall survival in our HCC cases could be due to its association with an extensive disease burden and therefore worse short-term mortality. On the other hand, the overall mortality of HCC cases may be also affected by the efficacy of several therapeutic interventions, which is not expected to be associated with any cfDNA marker. In our study, the BCLC stage was not associated with patients’ overall survival. This could be attributed to the fact that our patients were almost exclusively classified as stage B.

Serum levels of the Alu247 element were higher in our HCC cases compared to matched CHB controls. Such a finding is in agreement with findings in patients with other malignancies who have been reported to have longer circulating Alu sequences of cfDNA more frequently compared to healthy individuals [14,15]. Circulating cfDNA consists of both coding and non-coding elements, including transposable repeated sequences, such as Alu repeats [11], and cancer is frequently related to a state of necrotic inflammation leading to a vast release of DNA fragments in the circulation [13,15]. 

Recent studies have demonstrated raised concentrations of serum cfDNA in patients with cancer [10,12,20]. In our study, however, there was no difference in the serum levels of cfDNA, as indicated by the housekeeping gene GAPDH and the short Alu repeat of 115 bp between HCC cases and CHB controls. In addition, these biomarkers were not associated with either the overall or one-year mortality of our HCC cases. Of note, both our HCC cases and controls suffered from CHB, which represents a chronic inflammatory hepatic disease, and as such, it is related to excessive release of DNA fragments in the bloodstream from non-cancerous hepatocytes [21].

Interestingly, circulating nucleosomes did not differ between our CHB patients with and without HCC. Nucleosomes were previously related to tumor detection and monitoring treatment response in oncology [17]. Our findings may be at least partially explained by the fact that circulating nucleosomes can be elevated in CHB irrespectively of HCC [21], while their levels may not increase further in CHB patients who develop HCC.

Our study was based on a homogeneous population of Caucasian patients with HBeAg-negative CHB, including cases with confirmed HCC and carefully matched controls who did not develop HCC for ≥5 years of follow-up. However, it has some limitations. Since all our patients were Caucasians, our results cannot be generalized in Asian CHB patients, in whom the pathogenesis of HCC may be different. Most importantly, it was a single-center study with a relatively limited number of participants, which prevented us from definitely excluding type II errors. In addition, we included HCC cases mostly of the intermediate stage (BCLC B), and thus our results need to be assessed in larger numbers of HCC cases of various stages. Therefore, further cohorts with larger sample sizes are required to validate our findings.

## 4. Materials and Methods

### 4.1. Patient Population

In total, 57 Caucasian adult patients with HBeAg-negative CHB were included. All patients were followed up at the outpatient liver clinics of the General Hospital of Athens “Laiko” over the last 5 years. In particular, 19 consecutive such patients who developed HCC were included (HCC cases) if they had available stored serum samples drawn within the first month after HCC diagnosis. Subsequently, another 38 matched CHB patients who did not develop HCC during ≥5 years of therapy with entecavir (ETV) or tenofovir disoproxil fumarate (TDF) were included (CHB controls) if they had an available serum sample drawn before ETV/TDF onset. CHB controls were selected at a 2:1 ratio with HCC cases after matching for the following main HCC risk factors in Caucasian, treated CHB patients: age (±5 years), gender, and platelet group (<100,000, 100,000–199,999, ≥200,00/mm^3^) [22]. Exclusion criteria for both HCC cases and CHB controls included decompensated cirrhosis; coinfection(s) with hepatitis D, hepatitis C, or human immunodeficiency virus, and a history of liver transplantation. Informed consent was obtained from all patients for the anonymous use of their data and biological material. The study protocol was approved by the university ethics committee.

### 4.2. Definitions and Follow-Up 

HBeAg-negative CHB was diagnosed in patients who had positive HBsAg and negative HBeAg for ≥6 months, elevated ALT levels on ≥2 monthly occasions, and serum HBV DNA > 2000 IU/mL. The severity of liver disease was determined before the onset of ETV/TDF. Only CHB without cirrhosis was diagnosed by compatible liver histological lesions, while CHB with cirrhosis (always compensated) was diagnosed by compatible histological, ultrasonographic, and/or endoscopic findings. In particular, the diagnosis of cirrhosis was based on histological findings in 20 of the 24 cirrhotic patients (8 with HCC and 12 controls) and on ultrasonographic (nodular liver parenchyma) and endoscopic (esophageal varices) findings in the remaining four patients. The HCC diagnosis was based on standard histological and/or imaging findings [1].

All CHB controls were followed according to the national guidelines. Physical examination and laboratory routine tests including, hematological and biochemical parameters, were obtained every 3–6 months. Serum HBV DNA levels were determined every 6–12 months. HCC surveillance was based on 6-monthly ultrasound imaging. HCC stage was diagnosed according to BCLC staging classification [1].

### 4.3. Laboratory Analysis

Serum samples were stored in polypropylene tubes at –80 °C until our analysis. All samples were collected within the first month after HCC diagnosis but before any treatment for HCC in HCC cases and before the onset of ETV/TDF in CHB controls. Some of our CHB controls were also included in a previous study by our group [23], in which, however, different serum samples at ETV/TDF onset were analyzed. 

The Plasma/Serum Cell-Free Circulating DNA Purification Mini Kit (cat. no. 55100) (Norgen Biotek. Corporation, Thorold, ON, Canada) was used to isolate circulating cfDNA from 200 μL serum samples [24]. For each sample, the concentration of the extracted cfDNA was measured by quantitative real-time polymerase chain reaction (RT-PCR) assay for the GAPDH housekeeping gene. The relevant primers used were GAPDHf: 5′-GGAAGGTGAAGGTCGGAGTC-3 and GAPDHr: 5′-GAAGATGGTGATGGGATTTC-3 [19,21]. All samples were analyzed in duplicate. The final value used was the mean of the two measurements.

The genomic equivalents (GE) of Alu115 and Alu247 repeats were assessed by quantitative RT-PCR. The relevant primers were 5-GGAGGCTGAGGCAGGAGAA-3 and 5-ATCTCGGCTCACTGCAACCT-3 for Alu115 as well as 5-CAAGACTTAGTACCTGAAGGGTGAA-3 and 5-CTTGCCTCTTTCCTAGCACTG-3 for Alu247, as forward and reverse, respectively [11,23].

TaqMan Control Genomic DNA (Applied Biosystems™ cat. no. 4312660) (human) was used to obtain the standard curve of cfDNA concentration and assess the GE of Alu115 and Alu247. One GE was considered equal to 3.3 pg of human genomic DNA [25].

BIORAD iQ5 with 45 cycles of amplification under standard cycling conditions (950 C for 1 min, 950 C for 3 s, and 600 C for 30 s) was used in order to conduct the quantitative PCR assays. Data collection and real-time analysis were enabled at 65 °C–95 °C for 30 s.

The master mix that was used for PCR assays was Kapa SYBR Fast qPCR Master Mix (Kapa Biosystem cat. no. KK 4608). 

Serum nucleosomes levels were measured by Cell Death Detection ELISA^PLUS^ (Roche Diagnostics, Burgess Hill, UK, cat. no. 11774425001) following the manufacturer’s instructions [26]. The results are expressed relative to controls, arbitrary units, or ng/mL.

### 4.4. Statistical Analysis

Statistical analyses were performed using SPSS (SPSS 26, IBM company, Chicago, IL, USA, 2019). All quantitative variables were presented as median values (interquartile range (IQR)). Quantitative variable comparisons between two groups were performed using the non-parametric Mann–Whitney test. The associations between quantitative variables were assessed by Spearman’s correlation and expressed by Spearman’s coefficient (r). Categorical variables were summarized as frequencies and percentages, and their associations were determined using the corrected chi-squared or two-sided Fisher’s exact test. Cumulative probabilities of HCC occurrence were estimated by Kaplan–Meier and compared using the log-rank test. To identify prognostic HCC risk factors, univariable and multivariable Cox proportional hazards models were used, and HRs with 95% CIs and *p* values were provided. Statistical significance was set at *p* < 0.05.

## 5. Conclusions

In conclusion, our case control study presents a novel understanding of the significance of serum circulating biomarkers in CHB-induced HCC. In particular, elevated levels of the element Alu247 and higher DNA integrity were observed in CHB patients who developed HCC than in CHB patients who did not develop HCC during ≥5 years of oral antiviral therapy with ETV or TDF.

## Figures and Tables

**Table 1:** Baseline characteristics of HBeAg-negative chronic hepatitis B patients with and without hepatocellular carcinoma (HCC cases and CHB controls).

Patient Characteristic	HCC Cases (N = 19)	CHB Controls (N = 38)	*p*
Age, years	71 (15)	71 (14)	0.63
Male gender, n (%)	17 (89.5)	35 (92.1)	0.74
Body mass index, kg/m^2^	25.1 (3.2)	25.6 (5.2)	0.52
Alcohol use, n (%)			0.61
None/mild (<20 g/24 h)	1 (66.7)	26 (70.3)	
Moderate	3 (16.7)	8 (21.6)	
Abuse (past or present)	3 (16.7)	3 (8.1)	
Smoking habits, n (%)			0.42
Never	13 (72.2)	20 (54.1)	
Past	2 (11.1)	8 (21.6)	
Current	3 (16.7)	9 (24.3)	
Diabetes mellitus, n (%)	1 (5.6)	9 (23.7)	0.10
Normal ALT (≤40 IU/L), n (%)	2 (13.3)	15 (44.1)	0.04
ALT in cases with elevated ALT, IU/L	134 (173)	112 (141)	0.47
Platelet counts, ×10^3^/mm^3^	143 (63.5)	151 (61.3)	0.48
Platelet groups, n (%)			0.45
≥200,000/mm^3^	3 (17.6)	3 (7.9)	
100,000–199,999/mm^3^	12 (70.6)	27 (71.1)	
<100,000/mm^3^	2 (11.8)	8 (21.1)	
Undetectable HBV DNA, n (%)	9 (47.4)	20 (52.6)	0.71
Cirrhosis, n (%)	10 (52.6)	14 (36.8)	0.26
For patients with cirrhosis			
Albumin, g/dL	3.6 (0.2)	3.6 (0.5)	0.23
Bilirubin, mg/dL	1.0 (0.2)	1.1 (0.3)	0.19
International normalized ratio	1.2 (0.2)	1.1 (0.2)	0.26
Child-Pugh score	5 (0)	5 (0)	0.84
PAGE-B score	19.7 ± 3.2	20.5 ± 3.0	0.38
HCC risk by PAGE-B score, n (%)			1.00
Low (PAGE-B score < 10)	0	0	
Medium (PAGE-B score 10–17)	4 (21)	8 (20.5)	
High (PAGE-B score > 17)	15 (79)	31 (79.5)	
Antiviral therapy, n (%)			
Entecavir	5 (26.3)	7 (18.4)	
Tenofovir disoproxil fumarate	14 (73.7)	31 (81.6)	

Quantitative variables are expressed as median values (interquartile range).

**Table 2:** Distinct circulating cell-free DNA (cfDNA) species in HBeAg-negative chronic hepatitis B patients with and without hepatocellular carcinoma (HCC cases and CHB controls).

Circulating cfDNA Species	HCC Cases (N = 19)	CHB Controls (N = 37)	*p*
cfDNA concentration, ng/mL	36.0 (94.0)	19.5 (27.3)	0.06
Alu115 levels, GE	50.1 (68.2)	33.9 (33.0)	0.14
Alu247 levels, GE	64.2 (67.9)	23.2 (29.7)	0.01
Alu247/Alu115 ratio (DNA integrity)	1.0 (0.7)	0.7 (0.3)	<0.001
Alu247/Alu115 ratio >1, n (%)	11 (57.9)	6 (15.8)	0.01
Total nucleosomes, ng/mL	25.2 (33.1)	26.4 (40.1)	0.78

GE: genomic equivalents. Quantitative variables are expressed as median values (interquartile range).

**Table 3:** Baseline characteristics of 57 HBeAg-negative chronic hepatitis B patients with or without hepatocellular carcinoma in relation to the presence of increased DNA integrity (Alu247/Alu115 ratio >1).

Patient Characteristic	Increased DNA Integrity		*p*
	Yes (N = 17)	No (N = 40)	
Age, years	71 (15)	70 (14)	0.35
Male gender, n (%)	14 (82.4)	38 (95)	0.12
Body mass index, kg/m^2^	25.0 (3.7)	25.6 (5.4) (3.7)	0.24
Alcohol use, n (%)			0.13
None/Mild (<20 g/24 h)	10 (66.7)	28 (70)	
Moderate	5 (33.3)	6 (15)	
Abuse (past or present)	0 (0)	6 (15)	
Smoking habits, n (%)			0.34
Never	11 (73.3)	22 (55)	
Past	1 (6.7)	9 (22.5)	
Current	3 (20)	9 (22.5)	
Diabetes mellitus, n (%)	2 (12.5)	8 (20)	0.51
Normal ALT (≤40 IU/L), n (%)	5 (41.7)	12 (32.4)	0.56
ALT in cases with elevated ALT, IU/L	62 (115)	59 (123)	0.92
Platelet counts, × 10^3^/mm^3^	145 (47)	151 (85)	0.62
Platelet groups, n (%)			0.06
≥200,000/mm^3^	1 (6.7)	5 (12.5)	
100,000–199,999/mm^3^	14 (93.3)	25 (62.5)	
<100,000/mm^3^	0 (0)	10 (25)	
Undetectable HBV DNA, n (%)	11 (64.7)	18 (45)	0.17
Cirrhosis, n (%)	8 (47.1)	16 (40)	0.62
PAGE-B score	19.7 (2.4)	20.6 (3.2)	0.45
HCC risk by PAGE-B score, n (%)			0.91
Medium (PAGE-B score 10–17)	3 (21.4)	8 (20)	
High (PAGE-B score > 17)	11 (78.6)	32 (80)	

Quantitative variables are expressed as median values (interquartile range).

**Table 4:** Cox regression analyses for associations of patients’ baseline characteristics and distinct circulating cell-free DNA (cfDNA) species with overall and one-year mortality in 19 HBeAg-negative chronic hepatitis B patients with hepatocellular carcinoma.

	Overall Mortality			One-Year Mortality		
	HR	95% CI	*p*	HR	95% CI	*p*
Age, per year	0.98	0.91–1.06	0.67	1.02	0.91–1.14	0.78
Gender, female vs. male	4.86	0.88–26.95	0.07	2.47	0.27–22.41	0.42
Body mass index, per kg/m^2^	0.90	0.69–1.17	0.43	0.74	0.44–1.25	0.26
Alcohol use, abuse vs. none/moderate	0.76	0.20–2.89	0.69	0.04	0–560.31	0.50
Smoking habits, current vs. never/past	0.81	0.18–3.69	0.78	0.04	0–560.31	0.50
Diabetes mellitus, yes vs. no	3.47	0.39–31.11	0.27	3.72	0.42–33.36	0.24
Normal ALT (≤40 IU/L), no vs. yes	0.39	0.05–3.21	0.38	0.33	0.03–3.69	0.37
Platelet counts, per 10^6^/mm^3^	1.00	0.99–1.01	0.37	1.00	0.99–1.01	0.35
Undetectable HBV DNA, no vs. yes	0.93	0.32–2.75	0.90	0.01	0–16.43	0.22
Alpha-fetoprotein, per ng/mL	1.00	1.00–1.00	0.11	1.00	1.00–1.00	0.52
Cirrhosis, no vs. yes	0.64	0.22–1.87	0.42	0.01	0–18.09	0.24
PAGE-B score, per unit	0.84	0.69–1.03	0.09	0.95	0.67–1.36	0.79
BCLC stage	1.04	0.42–2.57	0.94	1.00	0.17–6.05	1.00
cfDNA concentration, per ng/mL	0.99	0.98–1.01	0.27	1.01	0.99–1.02	0.36
Alu115 levels, per GE	0.99	0.98–1.00	0.06	0.99	0.97–1.01	0.28
Alu247 levels, per GE	0.99	0.98–1.01	0.07	0.99	0.98–1.01	0.58
Alu247/Alu115 ratio (DNA integrity), per unit	2.16	1.02–4.61	0.06	2.46	1.15–5.28	0.02
Nucleosomes, per ng/mL	0.98	0.96–1.01	0.18	1.01	0.98–1.05	0.44

HR: hazard ratio, CI: confidence interval, GE: genomic equivalent.

## Data Availability

Data available upon request.
